# Role of the Novel Peptide Phoenixin in Stress Response and Possible Interactions with Nesfatin-1

**DOI:** 10.3390/ijms22179156

**Published:** 2021-08-25

**Authors:** Tiemo Friedrich, Andreas Stengel

**Affiliations:** 1 Charité Center for Internal Medicine and Dermatology, Department for Psychosomatic Medicine, Charité—Universitätsmedizin Berlin, Corporate Member of Freie Universität Berlin, Humboldt-Universität zu Berlin and Berlin Institute of Health, 10117 Berlin, Germany; tiemo.friedrich@charite.de; 2 Department of Psychosomatic Medicine and Psychotherapy, University Hospital Tübingen, 72076 Tübingen, Germany

**Keywords:** anxiety, brain-gut axis, GPR173, phoenixin, nesfatin-1, stress

## Abstract

The novel peptide phoenixin was shown to be involved in several physiological processes ranging from reproduction to food intake. Interest in this protein has steadily increased over the last few years and its known implications have become much broader, playing a role in glucose homeostasis, anxiety, nociception, and pruritus. Phoenixin is expressed in a multitude of organs such as the small intestine, pancreas, and in the hypothalamus, as well as several other brain nuclei influencing numerous physiological functions. Its highly conserved amino-acid sequence amongst species leads to the assumption, that phoenixin might be involved in essential physiological functions. Its co-expression and opposing functionality to the extensively studied peptide nesfatin-1 has given rise to the idea of a possible counterbalancing role. Several recent publications focused on phoenixin’s role in stress reactions, namely restraint stress and lipopolysaccharide-induced inflammation response, in which also nesfatin-1 is known to be altered. This review provides an overview on the phoenixins and nesfatin-1 properties and putative effects, and especially highlights the recent developments on their role and interaction in the response to response.

## 1. Introduction

Phoenixin was first described in 2013 in its 14 and 20 amino-acid sequence forms as a reproductive peptide, modulating the expression of the gonadotropin-releasing hormone (GnRH) receptor [1]. It was discovered using a bioinformatic search for peptide sequences that are highly conserved across multiple species [1]. While a 17 amino-acid variation of phoenixin was mentioned [2] and a 15 amino acid variation (PNX14-Gly) is available for purchase [3], phoenixin in its 14 and 20 amino-acid isoforms are the most widely studied variants of the peptide [4] and phoenixin-14 is the most abundantly present isoform in rat spinal cord [5]. Although the physiological differences between phoenixin-14 and -20 are not yet fully elucidated, phoenixin-20 elicited a significant increase of luteinizing hormone (LH) at a dose of 100 nmol, while phoenixin-14 required a dose of 1000 nmol in a cell culture model [1], suggesting a higher biological activity of phoenixin-20. Its precursor is the uncharacterized protein C4orf52 [5,6] also known as “small integral membrane protein 20” (SMIM20) [4] and “mitochondrial translation regulation assembly intermediate of cytochrome c oxidase protein of 7 kDa” (MITRAC7) [6,7,8]. Phoenixin’s molecular weight is 1583.78 g/mol [9].

Nesfatin-1 was first described in 2006 as a satiety inducing peptide in the rat hypothalamus [10]. Similar to phoenixin, it was discovered employing a bioinformatic approach as a cleavage product of its highly conserved precursor nucleobindin-2 (NUCB2) [10]. NUCB2 is a protein consisting of 396 amino acids of which amino acids 1–82 are cleaved to nesfatin-1. Although other possible nesfatin molecules exist (namely nesfatin-2 cleaved from amino acids 85–163 and nesfatin-3 cleaved from amino acids 166-396), synthetic nesfatin-2/3 peptides did not affect appetite [10]. The biological role of these peptides remains unknown. The molecular weight of synthetic human nesfatin-1 is 9551.86 g/mol [11].

## 2. Expression Sites of Phoenixin and Nesfatin-1

In the initial paper describing phoenixin, the peptide was present most abundantly in the hypothalamus but also in the heart, thymus, stomach, and spleen. The authors proposed the pituitary gland as the primary physiological target of phoenixin [1]. Phoenixin was also shown to be expressed in the Edinger-Westphal nucleus (EW), the dorsal motor nucleus of the vagus nerve (DMN), and the nucleus of the solitary tract (NTS) [1]. The expression pattern did not differ between sexes [1]. We previously showed phoenixin immunoreactivity in the medial division of the central amygdaloid nucleus (CeM), the bed nucleus of the stria terminalis (BST), the supraoptic nucleus (SON), the arcuate nucleus (Arc), the raphe pallidus (Rpa), area postrema (AP), as well as the spinal trigeminal tract (sp5) and spinocerebellar tract [12]. We were also able to show expression of phoenixin in several peripheral tissues, namely the duodenum, ileum, jejunum, and pancreas [12], while we did not observe any immunoreactivity in the heart, thymus, stomach or spleen [12]. Phoenixin immunoreactivity was also shown in the paraventricular nucleus (PVN), lateral hypothalamus (LH), and ventromedial hypothalamus (VMH) [13], which we did not observe [12]. These discrepancies will have to be further studied.

NUCB2/nesfatin-1 is expressed in multiple tissues such as adipose tissue [14], X/A-like cells of the stomach [15,16], pancreas [17], specifically pancreatic beta-cells [18], testis [19], and the lung [20]. In the central nervous system, NUCB2/nesfatin-1 immunoreactivity was detected in various nuclei of the hypothalamus [21], piriform, cingulate, as well as insular cortex, medial preoptic area, ambiguus nucleus, nucleus accumbens (EW) [22], the bed nucleus of the stria terminalis (BST) [23], ventrolateral medulla, dorsal raphe nucleus and gigantocellular reticular nucleus, and cerebellum, as well as in autonomic sympathetic and parasympathetic preganglionic neurons of the spinal cord [21,24]. It should be noted that peripheral mRNA NUCB2/nesfatin-1 expression levels—mainly the stomach—are significantly higher than the expression in the central nervous system [16]. The NUCB2/nesfatin-1 co-expression with numerous peptides, namely oxytocin and vasopressin in the PVN and SON, cocaine-amphetamine-regulated transcript (CART) in the Arc and LH, tyrosine hydroxylase in the Arc and NTS, and melanin-concentrating hormone in the LH, as well as corticotropin-releasing factor (CRF), thyrotropin-releasing hormone (TRH), somatostatin, neurotensin, and growth-hormone-releasing hormone in parvocellular neurons, support the hypothesized pleiotropic functions of NUCB2/nesfatin-1 [22,25].

NUCB2/nesfatin-1 and phoenixin immunoreactivity overlap in numerous nuclei, namely the lateral septum, BST, as well as the CeM [12,21] and were shown to be co-expressed in the Arc (86% ± 6%), VMH (76% ± 7%), PVN (70% ± 6%), and LH (70% ± 8%) [13]. The overlap in expression suggests an interaction of the two peptides, which is further supported by their counterbalancing effects in several physiological processes as discussed below.

## 3. Receptors of Phoenixin and Nesfatin-1

In order to identify a possible phoenixin receptor out of the 150 orphan G-protein coupled receptors (GPCRs) for which no ligand has yet been identified [5], researchers used deductive ligand receptor matching and identified the G-protein coupled receptor 173 (GPR173) as a possible receptor [26]. Its importance for phoenixin messaging was proven by siRNA knockdown, which ameliorated the augmenting effects of phoenixin on GnRH-mediated LH release in cultured pituitary cells, as well as extending the estrous cycle [26]. Its expression was shown to be downregulated when phoenixin expression was increased after GnRH-R agonist or antagonist treatment [4]. Since GnRH also modulates the expression of its own receptor [27,28], this could suggest a negative feedback loop between phoenixin and GPR173 expression. Although this does not definitively prove GPR173 as “the” phoenixin receptor, it has been widely accepted as the most likely candidate.

Although the nesfatin-1 receptor is not yet described, there are several papers studying its properties. Nesfatin-1 interacts with a GPR receptor, leading to a calcium influx [22], which is suppressed by N- [29], L-, and P/Q-type calcium channel blockers [22], suggesting a mode of action involving these channels. Previous studies indicate that part of the signaling cascade is a G_i/o_ [22,30] and/or G_S_-protein-coupled receptor activation, as well as protein kinase A signaling [22]. Autoradiography indicated possible receptor expression sites in various endocrine organs such as the pituitary, adrenal glands, testis, pancreas, and adipose tissue, as well as skeletal muscle, liver, kidneys, heart, and lungs. Central putative receptor expression sites included the cortex, PVN, AP, DMN, and cerebellum [31], suggesting a broad homeostatic function of nesfatin-1.

## 4. Pleiotropic Effects

Initially, phoenixin-14 and -20 were shown to increase the effects of GnRH on LH release in cultures of female rat pituitary cells, namely by increasing the release of LH when cells were stimulated with GnRH, while not affecting the release of LH by itself. GnRH, produced by neurons in the hypothalamus, regulates the release of reproductive peptides such as LH and follicle-stimulating hormone (FSH) [32,33]. Phoenixin did, however, not affect the release of adrenocorticotropic hormone (ACTH), prolactin, growth hormone (GH) or TSH when stimulation of GnRH was absent [1]. Phoenixin also seemed to stimulate FSH mRNA expression, although results in that regard are unclear due to a similar increase caused by a control medium and only differed significantly when GnRH treatment was performed for 4 h, but not after 2 h [1]. Interestingly, this increasing effect of GnRH was not observed in male pituitary cell cultures [1]. The authors also proposed a pathway of phoenixin upregulating the GnRH receptor transcription, thereby increasing the GnRH effects, which was supported by an increase in GnRH receptor expression after treatment with phoenixin in female pituitary cells [1]. Disruption of physiological phoenixin signaling using siRNA altered the estrous cycle of rats, suggesting a direct influence of phoenixin on reproduction in females [1]. In humans, phoenixin serum levels were increased in female patients with polycystic ovary syndrome (PCOS) with significant positive correlations between phoenixin and LH, FSH and progesterone, as well as a negative correlation with estrogen [34].

Intrathecal administration of phoenixin at a dose of 1.25 and 2.5 μg was shown to reduce pain response behavior (writhes) in mice following an intraperitoneal (ip) injection of acetic acid, while the pretreatment with phoenixin antiserum increased the pain-related behavior [5]. Phoenixin being expressed mostly in medium to large neurons with a diameter of >25 μm [5] would, however, not support its proposed influence on nociception, since pain is mostly transmitted by neurons with a smaller diameter (<25 μm) [35,36,37].

Subcutaneous (sc) injection of high doses (4–16 mg/kg) of phoenixin-14 in the neck elicited a significant scratching response in mice within 5 min after injection lasting for up to 15 min, the greatest response was observed after injection of 8 mg/kg [2]. This effect was abolished by pretreatment with kappa-opioid agonist nalfurafine [2]. The observed pruritogen effect was, however, much less pronounced than for known pruritogens such as 5′guanidinonaltrindole (5′-GNTI), both in intensity and duration [2,38].

Phoenixin has a distinct orexigenic effect when applied intracerebroventricularly (icv) but not when applied peripherally, namely after ip injection [39]. After an icv injection, the light phase food intake was dose-dependently increased by 74% (1.7 nmol/rat) and 154% (15 nmol/rat). Moreover, the dark phase food intake was increased by 54% following an icv injection of the higher dose (15 nmol/rat) [39]. This was accompanied by an increased neuronal activity and expression of the peptide nesfatin-1 in several brain nuclei, namely the lateral septal nucleus, SON, PVN, as well as NTS [40]. Its involvement in food intake has been underlined further by a recent report suggesting an implication of phoenixin in glucose homeostasis: Phoenixin was found both in alpha as well as beta cells in the pancreas and its secretion was stimulated by glucose, while itself stimulating insulin mRNA expression in a beta cell culture model employing INS-1E cells [41]. Phoenixin also directly influenced insulinergic glucose response via cAMP/Epac signaling [41]. Taken together, this makes a physiological role of phoenixin in energy homeostasis likely.

Phoenixin was shown to be negatively associated with anxiety levels in obese men, while not being correlated with perceived stress or depressiveness [42]. Treatment with phoenixin by an icv injection had anxiolytic effects in mice [43]. Phoenixin-14 (25 nmol) injected icv or infused into the hippocampus increased the memory function in mice [44]. This effect depended on GnRH as shown by the pretreatment with the GnRH receptor antagonist cetrorelix [44]. The observed improvement of memory could potentially be of interest in the treatment of Alzheimer’s dementia, since phoenixin restored memory function after artificial impairment with scopolamine (ip) or Aβ1-42 (icv) [44]. In humans, plasma phoenixin levels were negatively correlated with logical memory function in patients with mild cognitive impairment [45]. Levels of plasma phoenixin were lower in patients with Alzheimer’s dementia compared to patients only subjectively complaining about memory impairment, but were interestingly higher than in patients with mild cognitive impairment, although these results did not reach significance [45]. Lastly, phoenixin’s precursor C4orf52 was previously shown to be deleted in patients who suffered from partial epilepsy with pericentral spikes [5], which can present with episodes of gastric pain [46], possibly resulting from a reduced release of phoenixin [5].

**Figure 1:**
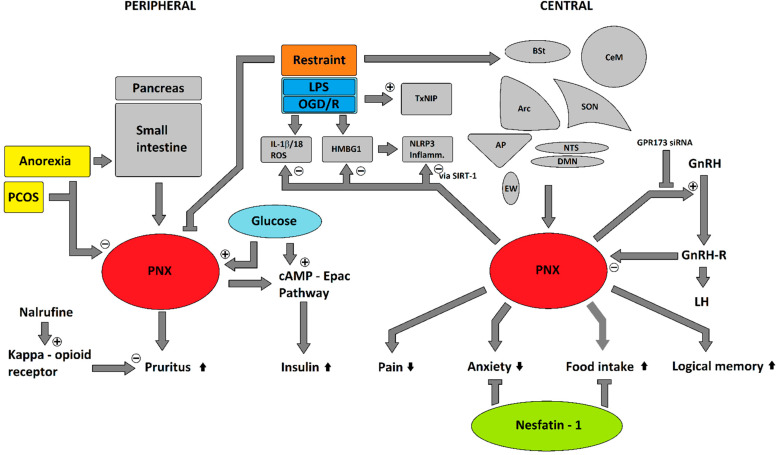
Phoenixin’s pleiotropic effects and interaction with nesfatin-1. Phoenixin influences various physiological processes such as metabolism, stress, hunger/appetite as well as the expression/serum levels of various chemokines/peptides. “+” or pointed arrowheads indicate an increase/augmentation of the targeted structure, “−“ or squared arrowheads indicate a decrease/inhibition of the targeted structure.

Similar to phoenixin, nesfatin-1 has effects on various physiological functions such as food intake [40,47], anxiety [48], duodenal [49] and gastric motility [47], influence on gonadotropins/sexual maturation [50,51], and on inflammation accompanied by reductions in TNF-α and interleukin levels, as well as an involvement in cyclooxygenase 2 signaling [52,53,54,55]. Nesfatin-1 potentially influences glucose homeostasis via glucose-stimulated insulin release in vitro and is colocalized with insulin in pancreatic beta cells [17,56], and plays a role in energy homeostasis as a whole by raising the body temperature after an icv injection [57]. The importance of endogenous NUCB2/nesfatin-1 was recently confirmed by an icv antibody injection in a rat model, which elicited an increase in food intake and a decrease in thermogenesis, proving that NUCB2/nesfatin-1 not only influences food intake and energy homeostasis pharmacologically but also at endogenous levels [58]. The NUCB2/nesfatin-1 effects on glucose metabolism are—at least partially—dependent upon the ghrelin receptor (growth hormone secretagogue receptor, GHSR) as recently shown through a murine knock-out model and GHSR antagonist co-injection with nesfatin-1, which attenuated (knock-out) or abolished (GHSR-antagonist) the nesfatin-1 effects [59]. Cardiovascular effects, namely an increase of blood-pressure in transgenic mice have also been reported [60] and are believed to be at least partially the result of cholinergic signaling [61]. Apart from homeostasis, nesfatin-1 has also been shown to influence neuropsychological processes such as anxiety and depression, namely an increase in anxious behaviour both after a central icv or peripheral ip injection in rodents [62,63]. In humans, circulating NUCB2/nesfatin-1 has also been associated with increased levels of anxiety [48]. Although nesfatin-1 is most strongly expressed in peripheral tissues [16], its ability to cross the blood-brain barrier allows peripheral nesfatin-1 to interact with central nervous signaling [64], thereby possibly eliciting the reactions that were previously described. The various physiological effects of phoenixin and its interaction with nesfatin-1 are summarized in Figure 1.

## 5. Potential Involvement in Stress Response

Converging evidence recently pointed towards an involvement of phoenixin in the response to stress. We showed a significant increase in central expression of phoenixin in rats subjected to restraint stress, a well-established emotional stressor, correlating with c-Fos activity in the DMN, mNTS and RPa [65], suggesting an involvement of phoenixin in the psychological stress response (Figure 1). The plasma concentration of phoenixin after restraint stress was significantly decreased 15 min after restraint stress and differed—although not significantly—until 60 min after restraint [66]. This coincided with an increase in serum cortisol levels, differing significantly compared to control animals after 30 min for the whole observation period of 4 h [66]. Phoenixin levels were, however, not correlated to cortisol levels [66]. The measured phoenixin serum levels were positively correlated with nesfatin-1 levels (r = 0.38) [66], further suggesting a possible interaction of these two peptides.

Apart from restraint stress, effects of phoenixin were also shown after immunological stress, namely the bacterial endotoxin lipopolysaccharide (LPS) (Figure 1) [67]. Phoenixin-20 decreased the activity of the NLRP3 inflammasome activation in a microglial cell culture [67]. This was accompanied by a reduction of ROS, IL-1β, and IL-18, which would suggest that phoenixin has anti-oxidative effects in microglia [67]. This is the result of a reduction in Thioredoxin-interacting protein (TxNIP) expression [67], which is required for NLRP3 inflammasome activation [68], thereby resulting in a neuroprotective effect [67]. This effect of phoenixin-20 on the NLRP3 inflammasome was dependent on the sirtuin-1 (SIRT-1) pathway, as shown by an amelioration when this pathway was inhibited by nicotinamide [67].

A similar effect was shown for phoenixin-14, which reduced LPS-induced endoplasmatic reticulum (ER) stress, thereby inhibiting the eukaryotic initiation factor-2 (eIF-2α), resulting in a reduced activation of transcription factor 4 (ATF4) and a reduced transcription of CCAAT/enhancer-binding protein-homologous protein (CHOP), a growth arrest and DNA damage-inducible protein 34 (GADD34) [69]. In addition, phoenixin-14 attenuated the LPS-induced increase in high mobility group box one (HMGB1) protein [69], which also induces the expression of NLRP3 [70]. This reduction in HMBG1 due to phoenixin-14 was also found in an oxygen-glucose-deprivation-reperfusion stress (OGD/R) cell culture model (Figure 1) [71].

A recent report studied phoenixin’s effects on NTS neuron excitability and by accident found stress to greatly influence phoenixin’s effects. After initially observing increases and decreases in spike frequency in NTS neurons after phoenixin treatment, this effect was abolished after construction started in their animal care facility [72], suggesting that stress might influence phoenixin’s effects on NTS neurons. To test this theory, the authors moved their animal facility to a new building, which restored their initial results. After further experiments, they were able to show that 22 days of corticosterone (CORT) pretreatment elicited the same effect in abolishing phoenixin’s effects on NTS neuron excitability compared to undisturbed rats [72]. Overall, the authors showed an increase in firing frequency in 32% of NTS neurons, as well as a depolarization after peptide application in 50% of the tested NTS neurons, thereby proving that phoenixin’s effects are influenced by psychological/environmental stress (i.e., construction noise) and endocrinological stress (i.e., CORT) [72]. They also hypothesized that this effect might be occurring due to a decrease in GPR173 expression [72].

During reperfusion stress in a cell culture stroke model, phoenixin-14 was shown to reduce the production of reactive oxygen species (ROS) after OGD/R [71], thereby reducing neuronal cell damage and inflammation caused by ROS [73]. This was attributed to a decrease in NADPH-Oxidase-1 (NOX-1) expression, which is a ROS producer, due to phoenixin-14 [71]. As previously mentioned, another effect was a reduction in HMBG1 expression, which induces the inflammatory response after cell injury [71]. Another effect in this model was a decrease in occludin expression after OGD/R, which is an important protein for blood-brain barrier function, that was ameliorated by phoenixin-14 [71]. It could be speculated, that one reason for phoenixin’s proposed inability to increase food intake when injected peripherally [39] is due to its own effects of tightening the blood-brain barrier.

Lastly, anorexia nervosa might be viewed as a metabolic stressor, where phoenixin levels were decreased in patients who suffered from this condition [74]. The most significant decrease was observed in patients with acute anorexia nervosa where phoenixin levels were positively correlated with BMI, while phoenixin levels were less markedly decreased in patients with anorexia nervosa who already regained body weight [74]. This reduction in phoenixin could speculatively be due to hypocaloric stress [65]. Moreover, anorexia nervosa is also often accompanied by increased cortisol levels [75] pointing towards an association with the hypothalamus-pituitary-adrenal [HPA] axis. In conclusion, phoenixin was shown to be both influenced by stress as well as being a potential modulator of stress. Since the peptide itself is novel and scientific interest in phoenixin is growing, its involvement in stress reactions will have to be studied further.

Similar to phoenixin, nesfatin-1 was also shown to be involved in various stress reactions such as restraint stress [76], surgical stress [77], and metabolic stress [78]. Nesfatin-1 can act as a direct activator of the HPA axis by stimulating an adrenocorticotropin as well as corticosterone plasma level increase after an icv application [79]. This reaction could potentially be the result of a direct receptor activation in the PVN as suggested by autoradiographic signals that showed a high concentration in this brain region [31]. The PVN is a pivotal region in the stress response, regulating neuroendocrine and cardiovascular reactions to stress, as well as being the main site of CRF neurons in the hypothalamus, which regulates ACTH release [80]. Its activation through stress also varies depending on the perceived controllability of stressors [81], leading to a significantly higher CRF neuron activity if the stressor is perceived as controllable [82]. The nesfatin-1 involvement in the response to stress is further supported by an observed increase in intracellular calcium levels after the nesfatin-1 treatment in isolated PVN neurons [83] and significantly increased activation of the PVN through an icv application of nesfatin-1 [83]. In light of the previously mentioned observed interaction of nesfatin-1 with GPRs leading to a cellular influx of calcium [22,29], the theory of nesfatin-1 receptors being present on PVN neurons and thereby possibly also reacting to peripheral nesfatin-1 signals is further supported. Nesfatin-1 immunoreactivity is also markedly increased in an abdominal-surgery-stress model, where the PVN, SON, LC, RPa, EW, as well as NTS and ventrolateral medulla showed a significant increase in neuronal activation as shown by increased c-Fos expression [77]. Restraint stress (30 min) showed a similar activation with increased immunoreactivity in the same areas except the EW [76] without altering plasma nesfatin-1 levels after 15 min of restraint stress [83]. Immobilization stress also increased NUCB2 mRNA expression in the PVN as well as the Arc [79,84] and induced an increased release of noradrenalin in the PVN as a result of signaling from the ipsilateral brainstem [85]. PVN activation through peripheral stress signals then leads to a release of ACTH from the pituitary, thereby activating the HPA [79]. Bilateral adrenalectomy led to an increase in NUCB2 mRNA levels suggesting a negative feedback loop with adrenal signaling [79]. A recent report showed that roughly one third of all nesfatin-1 positive neurons in the PVN as well as the Arc also express glucocorticoid receptors, which support the theory of an influence of peripheral adrenal stress signals on central NUCB2/nesfatin-1 expression [86]. Nesfatin-1 was also shown to be expressed in neurons of the BST in humans [23], which is known to be an important neuronal structure for anxiety [87], stress [88], and fear [89]. The volume of the central part of the BST is higher in men than in women [90,91]. This difference in volume coincides with the reported higher NUCB2/nesfatin-1 serum levels in women compared to men, with positive correlations to perceived stress and depressiveness in women, but not in men [92] The correlation in anxiety scores was inverse, showing a positive correlation in women, while being negatively correlated in men [92]. Since the icv injection of nesfatin-1 also induced an increased activity in nuclei that also express nesfatin-1, such as the PVN, SON, NTS, and LC [83], a paracrine mode of action could also be part of nesfatin-1 signaling [93]. Due to the nesfatin-1 ability to cross the blood-brain barrier [64], peripheral administration via the ip injection also evoked an acute central-nervous response with elevated CRF mRNA expression in the hypothalamus and an increased activity of the HPA axis leading to increased corticosterone plasma levels, as well as increased interleukin-6 (IL-6) and C-reactive protein (CRP) levels after chronic administration as part of an inflammatory reaction [94]. Although an increase in CRP would indicate pro-inflammatory properties, nesfatin-1 was also shown to suppress inflammation via an inhibition of NF-κB/NLRP3 inflammasome signaling and a reduction in HMBG1 expression, thereby protecting against oxidative stress and apoptosis [78]. In conclusion, nesfatin-1 neuron activity and NUCB2/nesfatin-1 expression have been shown to be influenced by various stressors. In addition, nesfatin-1 itself has been shown to have an influence on stress reaction and anxiousness, suggesting a complex involvement in subjective stress experience as well as physiological stress response.

## 6. Interactions

Phoenixin and nesfatin-1 show opposing effects in various physiological processes: The nesfatin-1 anorexic properties are—at least in part—mediated via leptin-independent oxytocinergic signaling [95], as well as by melanocortin signaling, since its effects were blocked by melanocortin-3/4 receptor antagonists [10]. This is in opposition to phoenixin which has strong orexigenic properties [39]. The phoenixin-induced stimulation of food intake was accompanied by an increase in nesfatin-1 immunoreactivity, suggesting a counterbalancing interaction of the two peptides [40].

These opposing effects have also been shown regarding anxiety—phoenixin exerting anxiolytic effects in mice [43], as well as being negatively associated with anxiety in humans [42], while nesfatin-1 increases anxiety-like behavior in rats [62] and being positively associated with higher anxiety in humans [48]. Similar results were seen after restraint stress, where phoenixin serum levels were decreased, while nesfatin-1 levels rose, although these effects were less pronounced [66]. In addition, both phoenixin and nesfatin-1 immunoreactivity were increased after restraint stress in the NTS and RPa in rodents, while showing a distinctly different pattern of expression in other nuclei [65,76], possibly modulating the relay of peripheral signals from baro- and osmoreceptors in the NTS to the PVN [96,97] and thereby, balancing physiological reactions to stress to avoid an excessive response.

This theory of phoenixin and nesfatin-1 interacting in a counterbalancing fashion was further corroborated by the discovery that both peptides are highly co-expressed in the hypothalamus, namely the ventromedial hypothalamus (VMH, 76%), Arc (86%), paraventricular nucleus (PVN, 70%), and lateral hypothalamus (LH, 70%) [13]. Apart from their expression in the central nervous system, nesfatin-1 and phoenixin-14 serum concentrations were also positively correlated in patients with PCOS [34], further suggesting a physiological interaction between the two peptides. A recent publication studied the effects of the long-term olanzapine treatment on both NUCB2/nesfatin-1 and phoenixin/SMIM20 mRNA expression in rodent brains and showed an increased expression of phoenixin/SMIM20 with an unaltered NUCB2/nesfatin-1 level, falling in line with the anxiolytic and antidepressant properties of olanzapine, as well as supporting the notion of a counterbalancing role of phoenixin and nesfatin in depression/anxiety [98].

## 7. Conclusions

Phoenixin was initially discovered as a reproductive peptide that has since then been shown to exert a wide array of functions. Since it is such a young peptide, our knowledge about its physiological functions in humans mostly relies on correlations and its mode of action is not yet clear. Nesfatin-1 is a new but extensively studied peptide, whose effects seem to oppose that of phoenixins in almost all bodily functions. The proposed link between nesfatin-1 and phoenixin and the influence they exert on their respective counterpart underlines the nesfatin-1 importance for a better understanding of phoenixin’s mode of action. The results that have been generated so far in rodent and cell culture models show promising results: Phoenixin’s orexigenic and anxiolytic/anti-depressive properties might bear therapeutic potential. Its role in glucose homeostasis and insulinotropic properties could speculatively make it a possible target for diabetes treatment. In stress reactions, phoenixin seems to be highly involved in several brain nuclei as well as—to some extent—also on a circulating level. Although stress itself is not a treatable disease, extreme stress responses and anxiety such as in patients suffering from posttraumatic stress disorder, as well as the increasingly prevalent subjective high stress levels in the general population, increase the attention on stress as an endemic problem and might further increase the interest in possible modulative treatments. This would make phoenixin a peptide with anxiolytic properties and possible attenuating effects on stress a possible candidate. Overall, the results generated over the last few years paint a promising picture, and interest in phoenixin is steadily increasing with its interactions with nesfatin-1 becoming more and more elucidated. While still in the early stages of research, phoenixin, its pathway, and possibly its counterpart, nesfatin-1, could further help our understanding of the psychoneurophysiology of stress.

## Data Availability

Not applicable.
